# Highly efficient THz four-wave mixing in doped silicon

**DOI:** 10.1038/s41377-021-00509-6

**Published:** 2021-04-01

**Authors:** Nils Dessmann, Nguyen H. Le, Viktoria Eless, Steven Chick, Kamyar Saeedi, Alberto Perez-Delgado, Sergey G. Pavlov, Alexander F. G. van der Meer, Konstantin L. Litvinenko, Ian Galbraith, Nikolay V. Abrosimov, Helge Riemann, Carl R. Pidgeon, Gabriel Aeppli, Britta Redlich, Benedict N. Murdin

**Affiliations:** 1 Radboud University, Institute for Molecules and Materials, HFML-FELIX, Nijmegen, The Netherlands; 2 Advanced Technology Institute and Department of Physics, University of Surrey, Guildford, GU2 7XH UK; 3 Institute of Optical Sensor Systems, German Aerospace Center, Berlin, Germany; 4 Institute of Photonics and Quantum Sciences, SUPA, Heriot-Watt University, Edinburgh, UK; 5 Leibniz-Institut für Kristallzüchtung (IKZ), Berlin, Germany; 6 Laboratory for Solid State Physics, ETH Zürich, 8093 Zürich, Switzerland; 7 Institut de Physique, EPFL, 1015 Lausanne, Switzerland; 8 Paul Scherrer Institute, 5232 Villigen, Switzerland

**Keywords:** Nonlinear optics, Silicon photonics, Terahertz optics

## Abstract

Third-order non-linearities are important because they allow control over light pulses in ubiquitous high-quality centro-symmetric materials like silicon and silica. Degenerate four-wave mixing provides a direct measure of the third-order non-linear sheet susceptibility *χ*^(3)^*L* (where *L* represents the material thickness) as well as technological possibilities such as optically gated detection and emission of photons. Using picosecond pulses from a free electron laser, we show that silicon doped with P or Bi has a value of *χ*^(3)^*L* in the THz domain that is higher than that reported for any other material in any wavelength band. The immediate implication of our results is the efficient generation of intense coherent THz light via upconversion (also a *χ*^(3)^ process), and they open the door to exploitation of non-degenerate mixing and optical nonlinearities beyond the perturbative regime.

## Introduction

**Fig. 1:**
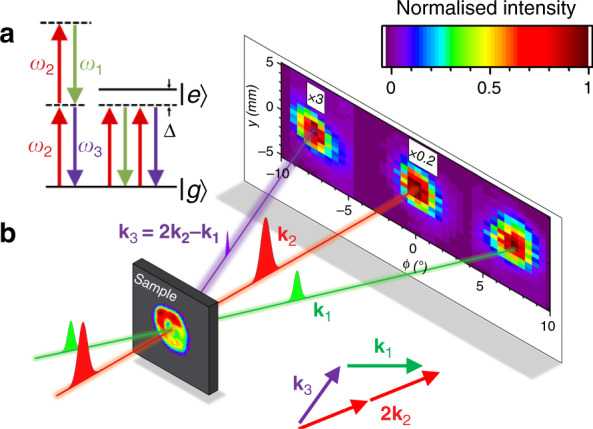
The degenerate four-wave mixing geometry. **a** The energy-level scheme for DFWM with two excitation photons from beam 2, a stimulated emission from beam 1 and an output photon in beam 3. The left hand process involves two virtual excited states, and the right hand permutation is the strongest near to a resonance with the ground state $\left|g\right\rangle$ and excited state $\left|e\right\rangle$. **b** A camera image of the beam at the sample position is shown superimposed on the sample. Far-field images were taken by scanning an iris after collimating, which requires careful conversion from space to angle. Each image has been normalised to the peak power density, and the scale factors for the far-field images are indicated relative to beam 1. Note that the far-field image of the beam 3 has only been scaled by a very small factor in this example (×3), i.e. the DFWM efficiency is very high. The phase matching condition is also shown

The lowest-order non-linearity in centrosymmetric materials is *χ*^(3)^, which describes that part of the response that is third order in the amplitude of the driving beams^1^. It is responsible for effects like degenerate four-wave mixing (DFWM), in which all four photons have the same energy and two are excited and two are emitted, Fig. 1. A substantial degenerate (or near-degenerate) FWM response is a prerequisite for applications of media in active optical systems ranging from modulators^2,3^ to quantum repeaters^4–6^.

Although many non-linear effects have been demonstrated in the THz domain^7^, there are no quantitative measurements of susceptibilities for transparent bulk materials—indeed there are very few values of *χ*^(3)^ available for any material in this part of the spectrum^8–11^.

Doped silicon at low temperature has already been shown to produce giant values for the imaginary part of the non-linear refractive index (via multi-photon absorption)^12^, and there have been theoretical predictions that the real part of the non-linear refractive index is also very large^13^ and of large experimental non-linearities^14^ but there have been no experimental reports of *χ*^(3)^ till now, largely because of the challenge of quantitative non-linear THz metrology.

## Results

### Experiment

We performed non-collinear DFWM experiments as illustrated in Fig. 1, using THz pulses from the free electron laser FELIX, both on and off resonance with the 1*s* → 2*p* transitions in Si:P and Si:Bi at 10 K. We chose this geometry because it enables the measurement of dynamical relaxation and dephasing times needed to make detailed theoretical comparisons, under identical experimental conditions. It is very difficult to obtain clean beam profiles with low diffraction in the THz regime, and great care was taken in avoiding apertures and optical imperfections in order to obtain them, as shown in Fig. 1. Care was also taken to accurately calibrate absolute pulse energies. It may be seen immediately from the relative strength of the output beam (**k**_3_) in Fig. 1 that the DFWM process is very efficient.

In the plane-wave limit (i.e., for infinitely long pulses and infinitely broad beams), the complex polarisation amplitude of the generated beam (${{\mathcal{P}}}_{3}$) is related to the complex field amplitudes of the input beams (${{\mathcal{F}}}_{1,2}$) inside the material by

1 $${{\mathcal{P}}}_{3}={\epsilon }_{0}{\chi }^{(3)}{{\mathcal{F}}}_{1}^{* }{{\mathcal{F}}}_{2}^{2}$$

i.e. the intensity of the output is determined by *χ*^(3)^. The definition of *χ*^(3)^ in Eq. (1) suggests that, for a pulsed experiment, the internal pulse energies *E*_*i*_ of the three beams **k**_*i*_ (Fig. 1) are related by

2 $${E}_{3}={E}_{1}{E}_{2}^{2}/{E}_{{\rm{c}}}^{2}$$

where *E*_c_ is a constant that is inversely proportional to *χ*^(3)^*L* and *L* is the sample thickness. *E*_c_ defined by Eq. (2) is a critical pulse energy at which the output would become equal to the inputs, and we generally stay well below this limit so as to avoid the need to consider higher-order non-linear effects.

**Fig. 2:**
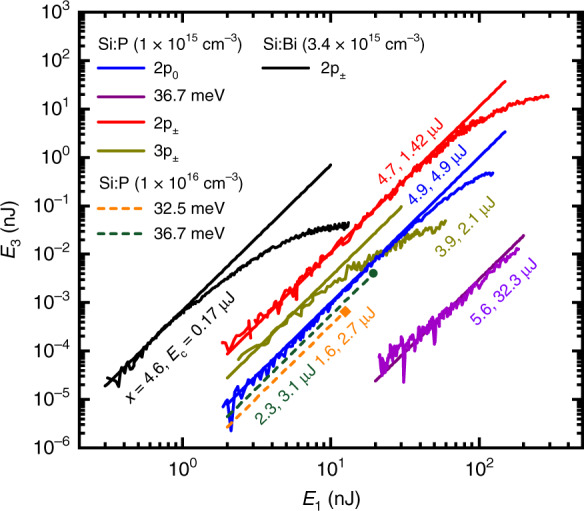
Internal DFWM conversion efficiency for different samples, both on and off resonance. The different doping densities (*n*_D_) and samples are given in the legend. Each curve is labelled by either the laser photon energy ($\hbar$*ω* in meV) or the resonant transition being excited. The ratio between pump pulse energies *x* = *E*_2_/*E*_1_ was kept constant in each case: values of *x* are given on each data set. The data are very close to cubic (${E}_{3}={x}^{2}{E}_{1}^{3}/{E}_{{\rm{c}}}^{2}$ as expected), and the solid lines are fits to the low intensity portion. The fitted values of *E*_c_ are also indicated. For the high density Si:P sample, only one intensity was measured at each laser frequency and a cubic dependence (dashed lines) is shown for comparison with the other measurements

We varied *E*_1_ keeping the ratio *E*_2_/*E*_1_ fixed, as shown in Fig. 2, and a clear cubic dependence is observed at low pulse energy. The resulting values of *E*_c_ are shown on Fig. 2 and given in Table 1. At high intensity, a saturation occurs for resonant cases, due to an intensity-dependent reduction in dephasing time^15^, which reduces *χ*^(3)^.

**Table 1:** Third-order susceptibility for Si:P and Si:Bi both on and off-resonance

	Si:P						Si:Bi
$\hbar$*ω* (meV)	32.5	34.0	36.7		39.2	42.5	64.5
	–	(2p_0_)	–		(2p_±_)	(3p_±_)	(2p_±_)
	T	R	T	T	R	R	R
*L* (mm)	0.6	0.5	0.5	0.6	0.5	0.5	1
*n*_D_	10	1.0	1.0	10	1.0	1.0	3.4
*x*	1.6	4.7	5.6	2.3	4.6	3.9	4.9
*r*_0_ (mm)	0.53	0.6	0.6	0.53	0.6	0.6	0.64
*E*_c_ (μJ)	2.7	4.9	32.3	1.1	1.4	2.1	0.17
*f*	3.0	28	6.1	6.2	310	27	310
${\chi }_{\,\text{expt}\,}^{(3)}L$	0.13	0.80	0.025	0.58	27	1.5	160
${\chi }_{\,\text{expt}\,}^{(3)}$	0.22	1.6	0.050	0.96	54	2.9	160
${\chi }_{\,\text{expt}\,}^{(3)}/{n}_{{\rm{D}}}$	0.022	1.6	0.050	0.096	54	2.9	46
*μ*_eg_ (*e*.nm)	–	0.37	–	–	0.71	0.32	0.34
$\hbar$/*T*_1_ (μeV)	–	11	–	–	5	3.9^a^	19
$\hbar$/*T*_2_ (μeV)	–	26	–	–	26	109	44
$\hbar /{T}_{2}^{* }$ (μeV)	–	115	–	–	115^b^	194	165
${\chi }_{\,\text{theory}\,}^{(3)}/{n}_{{\rm{D}}}$	0.0024	100	0.015		3100	23	18

$\hbar$*ω* is the photon energy, and labels R and T refer to resonant and transparent excitations. Values of *μ*_eg_ are all taken from ref. ^29^. All values for *T*_1,2_ were found from photon echo and pump–probe performed under the DFWM conditions, except: ^a^taken from ref. ^21^. All values of the half-width, $\hbar /{T}_{2}^{*}$, were found from the small-signal absorption spectrum, except: ^b^assumed equal to the 2p_0_ half-width. *x* is the ratio of the intensities of the pump pulses from Fig. 2. *L* is the sample thickness and *r*_0_ is the spot size. The dimensionless factor *f*, which is unity for zero loss and infinitely long pulses, appearing in Eq. (3) (and described in detail in the text), was found from integrating the propagation equations. The experimental values of *E*_c_ were extracted from Fig. 2. Values of *n*_D_ are given in units of 10^15^ cm^−3^; *χ*^(3)^*L* in units of 10^−16^ m^3^ V^−2^; *χ*^(3)^ in units of 10^−13^ m^2^ V^−2^; and *χ*^(3)^/*n*_D_ in units of 10^−34^ m^5^ V^−2^. Theoretical predictions are from Eq. (4), and for off-resonance excitation at 36.7 meV, the 2p_±_ contribution was used (it has much higher ${\mu }_{{\rm{eg}}}^{4}$) while at 32.5 meV we used the 2p_0_ contribution (it has much smaller Δ)

### Conversion of *E*_c_ to *χ*^(3)^

Away from resonance and in the limit of long pulses, the relationship between *E*_c_ (given on Fig. 2) and *χ*^(3)^ has a straightforward dependence on the geometry and pulse duration. For short pulses, the dynamics are important, and on resonance there is loss that attenuates the pumps and the output, which must also be taken into account. We integrated the equations describing propagation of light through a lossy non-linear medium for the case of inhomogeneous broadening and finite pulse durations to find *χ*^(3)^ from *E*_c_ (see Supplementary Materials Section IV). In this case, the conversion from the experimental *E*_c_ of Fig. 2 to the value of *χ*^(3)^ is, for a beam with a Gaussian spatial profile,

3 $${E}_{{\rm{c}}}={3}^{3/4}\sqrt{2\pi }{n}^{2}{\lambda }_{0}{r}_{0}^{2}{t}_{0}f/{Z}_{0}{\chi }^{(3)}L$$

where *n* is the refractive index (which we took to be *n* = 3.4), *λ*_0_ is the free-space wavelength, *Z*_0_ is the characteristic impedance of free space, and *r*_0_ and *t*_0_ are the root mean square (r.m.s.) beam radius and pulse duration, respectively (at which the intensity has fallen by $1/\sqrt{e}$).

The dimensionless factor *f* appearing in Eq. (3) depends on the loss and also the pulse shape and duration relative to the dynamical timescales of the system. The equation defines *f* in such a way that *f* = 1 when the loss is negligible (which is our case when far from resonance) and in the monochromatic limit of very long pulses with Gaussian temporal profile (*t*_0_ ≫ *T*_1,2_, i.e. pulse duration much larger than the population decay, *T*_1_, and dephasing time, *T*_2_, of the system). For negligible loss but with pulses that are very short compared with the inverse line-width, then *f* becomes of order *T*_1_/*t*_0_, which can evidently be larger than unity (effectively replacing *t*_0_ in Eq. (3) with *T*_1_ because now the atomic polarisation ${{\mathcal{P}}}_{3}$ lasts much longer than the drive pulses). For significant loss, *f* becomes very large and sample thickness dependent.

Using perturbation theory for temporally overlapping, weak beams within the two-level approximation^1^, and averaging over the distribution for a Gaussian (fully inhomogeneously broadened) line, we calculated values of *f* for our experimental circumstances. See Supplementary Materials for more details. The results are shown in Table 1. As expected, the off-resonant values of *f* in Table 1 are of order unity and are not significantly affected by the details of the model chosen. They are slightly greater than unity primarily because of the short pulses. The on-resonance values of *f* in Table 1 are large primarily because of the loss. The two-level model is expected to give a reasonably good estimate of *f* in resonant cases because there is one dominant transition: the one shown in Fig. 1a^1^.

The experimental values of *E*_c_ from Fig. 2 along with the calculated *f* have been converted to values of ${\chi }_{\,\text{expt}\,}^{(3)}$ in Table 1.

### Theory

We now obtain theoretical estimates for *χ*^(3)^ to compare with the experimental results. Silicon donors at low temperature are hydrogen-like, with a series of levels and orbitals closely resembling the Rydberg series 1s, 2p_0_, etc.^16^. The energies are scaled down and the orbital sizes scaled up, thanks to the small effective mass and large dielectric constant. The large orbitals give a commensurately large dipole moment, and this has a very large influence on non-linear optical coefficients.

Using the same two-level model mentioned above, the following limits may be found (see Supplementary Materials) for the contribution per bound electron in the vicinity of its resonance:

4 $$\frac{{\chi }^{(3)}}{{n}_{{\rm{D}}}}\approx \frac{{\mu }_{{\rm{eg}}}^{4}}{{\epsilon }_{0}{\hbar }^{3}}\times \left\{\begin{array}{ll}{T}_{1}{T}_{2}{T}_{2}^{* },&\,\text{if}\,\ {{\Delta }}=0\\ {T}_{1}{T}_{2}^{-1}{{{\Delta }}}^{-3},&\,\text{if}\,\ | {{\Delta }}| \gg {T}_{2}^{* -1}\end{array}\right.$$

where *n*_D_ is the donor concentration, $\hbar$Δ is the detuning from resonance in energy and ${\mu }_{{\rm{eg}}}=e| \langle {\psi }_{e}|{\bf{r}}|{\psi }_{g}\rangle .{\boldsymbol{\epsilon }}|$ is the component of the dipole moment transition matrix element between ground and excited states along the polarisation direction, *ϵ*. The total dephasing time ${T}_{2}^{* }$ is defined by the total absorption line half-width in energy, $\hbar /{T}_{2}^{* }$, which was obtained from the small-signal absorption spectrum. The population relaxation time, *T*_1_, was obtained by performing a pump–probe experiment^17^, and the homogeneous dephasing time, *T*_2_, was obtained using a photon echo experiment^1,15^. The results are shown in Table 1. These time-resolved experiments were performed with the same set-up that was used for the main DFWM experiment, simply by varying the delay between the beams and changing the position of an iris after the sample. This ensures that times *T*_1,2_ were obtained under the same experimental conditions as Fig. 2. The calculated values of *χ*^(3)^/*n*_D_ in the approximation of Eq. (4) are shown in Table 1 as ${\chi }_{\,\text{theory}\,}^{(3)}/{n}_{{\rm{D}}}$. These predictions from the two-level model may be expected to give reasonable order of magnitude estimates, but it should be noted that the intermediate states and permutations neglected in the approximation of Eq. (4) can give both positive and negative contributions. Earlier work on theoretical prediction of *χ*^(3)^ for silicon donors has included an infinite number of all possible intermediate states but not the dephasing and decay (*T*_1_, *T*_2_ and ${T}_{2}^{* }$)^13^.

## Discussion

In the transparent regions, away from resonance (labelled T in Table 1), we obtain very good agreement between experiment and theory; there is also suitable but not perfect scaling with impurity density, *n*_D_. The approximate theory in Eq. (4) consistently underestimates the experiment by about an order of magnitude, implying that the neglected terms due to higher intermediate states and other permutations are additive. On resonance (R), the agreement is almost as good, with a similar magnitude of discrepancy but this time in either direction (particularly notable when we compare 2p_±_ transitions for P and Bi), presumably because of the strong sensitivity to the effect of the loss. It is obvious that resonance significantly reduces *E*_c_ in Fig. 2 and enhanced *χ*^(3)^ relative to the non-resonant cases.

**Fig. 3:**
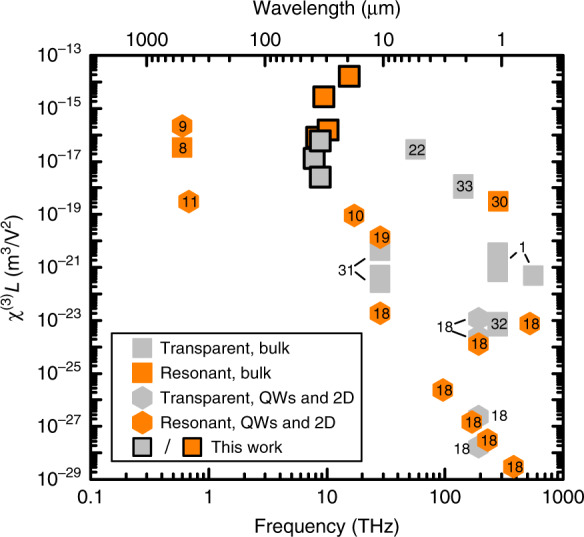
Survey of coherent values of *χ*^(3)^*L* from this work (symbols with solid borders) and elsewhere. Experiments where the pump transition is virtual are labelled as “Transparent”, while experiments with real pump transitions and consequent absorption losses have been labelled as “Resonant”. Resonant quantum wells^9,19^; graphene^10,11^ and ref. ^18^ and references therein; free-carrier processes in Si^8^; resonant bandgap-pumped bulk Si^30^; transparent 2D materials (MoS_2_ and black phosphorus) ref. ^18^ and references therein; transparent bulk semiconductors and insulators^22,31–33^ and ref. ^1^ (Table 4.6.1) and references therein

Figure 3 shows a survey of coherent *χ*^(3)^ measurements in other materials, systems and frequency bands. The figure shows *χ*^(3)^*L* since this is the quantity that has actually been measured in each case, and it is the quantity that is relevant for frequency mixing applications. In Fig. 3, experiments in which the pump transition is virtual have been labelled as transparent, and those where there is a real absorption process at the pump frequency have been labelled as resonantly enhanced. For example, free carrier processes can produce not only a very large *χ*^(3)^*L*^8,9^ but also very significant absorption; Dirac materials like graphene produce large *χ*^(3)^*L*^11,18^ but have resonant interband or free-carrier processes depending on the chemical potential; and resonant enhancements by quantum well design^19^ or Landau levels^10,20^ also naturally induce absorption pathways. In such cases (where absorption loss is present), the volume susceptibility *χ*^(3)^ is not an especially useful figure of merit for the material, because the output varies in a non-trivial way with sample thickness thanks to the loss. We note that very large apparent values of *χ*^(3)^ have been reported in two-dimensional and quantum well systems^9,11,19^. In all these cases, the measured output is normalised by the (very small) thickness, and the sheet susceptibility, *χ*^(3)^*L*, is very small by comparison with the values reported here and would remain so even for stacks of very many layers. It is interesting to note the general trend in Fig. 3 to increased susceptibility as the frequency is reduced. It can be seen from Eq. (4) that there is no intrinsic frequency dependence, so this increase is likely to be due to the difficulty of observing all but the strongest effects at THz frequency. It happens that the material used here has particularly large dipole moments^12^, which enter Eq. (4) with the fourth power, and an advantageous combination of long dephasing and decay times^15,21,17^.

Our measured *χ*^(3)^*L* far from resonance is a record for any transparent material, and the only such measurement for THz pumping. The *χ*^(3)^ values are all larger even than low temperature bulk InSb close to its band edge frequency^22^, meaning that here the contribution per electron (the hyperpolarisability) is far, far larger. This material can easily be produced in macroscopic thicknesses relevant for devices. An obvious immediate application is for metrology of a weak (**k**_1_) THz beam with an arrival time clocked by a strong coupling pulse (**k**_2_). Further prospects arise because compact and efficient semiconductor sources^23^ now cover the entire electromagnetic spectrum from radio waves to the ultraviolet with just one gap between about 5–15 THz (thanks to phonons in common polar solids): doped silicon could fill the gap by tripling the emission wavenumber for existing semiconductor THz lasers. For perspective, we point out that generation of THz light by downconversion from near-infrared (near-IR)^24–26^ and mid-IR^27^ sources is well established, but the efficiency is rather low, typically parts per thousand^24^. Our experiments were performed at cryogenic temperature, but many THz sources and detectors already require cryogenic environments^23^, and the operating temperature might be raised in future by exploiting deeper donors.

## Materials and methods

### Samples

Samples used were single-crystal silicon doped with either bismuth or phosphorous and kept at a temperature of 5–10 K during the experiment.

The donor densities were determined by four-point resistivity measurements. In all cases, the surfaces were chemically and mechanically polished with a wedge of about 1°. The small-signal absorption was measured with Fourier transform spectroscopy with the samples mounted in liquid helium at 2.2 K (see Supplementary Materials for absorption spectra), and the half-width of each inhomogeneously broadened transition, $\hbar /{T}_{2}^{* }$, was obtained from Gaussian fits. One transition, the 1*s* → 2*p*_±_ line in P-doped samples, was saturated, and we took the line-width to be the same as for the 1*s* → 2*p*_0_ line in this case. The concentrations, sample thicknesses and line-width values are given in Table 1.

### Dynamical measurements, beam imaging and overlap

The optical set-up was a standard, time-resolved, forwards DFWM arrangement^10^, Fig. 1. All beams were focussed into a cryostat, recollimated and refocused using off-axis parabolic mirrors onto a high sensitivity liquid He-cooled Ge:Ga photo-conductive detector with a response time of about 100 ns.

A mechanical moving stage controlled the delay, and for the photon echo experiment (used to measure *T*_2_), we measured the **k**_3_ beam pulse energy as a function of the delay between **k**_1_ and **k**_2_ beams, while for the pump–probe experiment we simply moved the iris to detect the transmitted **k**_1_ beam, which then functions as a weak probe. To ensure optimal overlap, we imaged the beams at the sample position with a pyroelectric camera with an effective pixel pitch of 80 μm (Spiricon Pyrocam IV). To obtain beam selection and optimal discrimination of the far-field beams after the sample, a motorised iris with a controllable aperture was mounted on a *x*–*y* scanning stage between the collimating mirror and the detector. The dependence of the output DFWM pulse energy shown on Fig. 2, *E*_3_, was measured with the iris open wide enough to capture the whole beam (while still excluding the pumps). The resulting *T*_1_ and *T*_2_ data are given in Table 1.

### Pulse energy calibration

For metrology of the pulses in each beam for the data of Fig. 2, we calibrated the photon energy-dependent responsivity of the detector before each measurement. As a reference standard, we used a calibrated pyro sensor (SLT PEM 34 IR) with an accuracy of 2%.

For each set of measurements, we determined the ratio *x* = *E*_2_/*E*_1_ by simultaneously recording both beams with the pyroelectric camera just before the sample, while scanning the undulator of the FEL.

The cryostat window transmission was calibrated by measuring the laser transmission through the empty cryostat (i.e. without the sample), referenced to the signal with the cryostat removed. We assumed both windows had the same transmission. The reflection loss at the sample surface was estimated using the Fresnel transmission coefficient ${\mathcal{T}}\approx 0.7$ for the interface of the sample, which approximately agrees with the laser transmission signal when very far from resonance.

A polariser pair (Infraspecs P03) before the beam splitter was used to adjust the total laser pulse energy in fine steps for the intensity dependence of Fig. 2.

The values of *r*_0_ used are given in Table 1 and came from the measurement with the Pyrocam mentioned above.

### Pulse shape

**Fig. 4:**
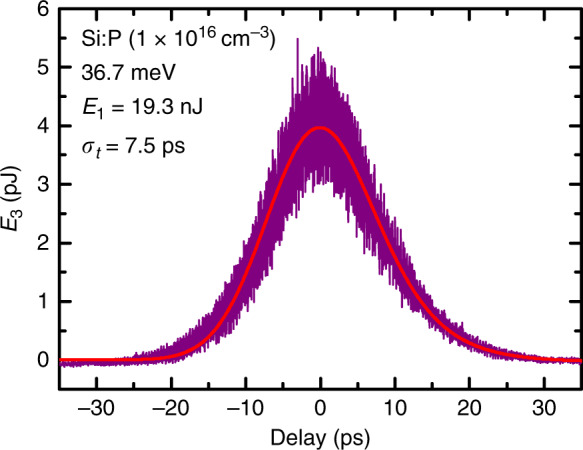
An example transient output pulse energy (*E*_3_) as a function of delay between the input pulses (the k_1_ and k_2_ beams) for one of the Si:P samples far from resonance. The signal is a third-order autocorrelation of the pulse temporal profile. This autocorrelation signal appears Gaussian to a good approximation (red line) with r.m.s. width *σ*_*t*_ = 7.5 ps, so the inferred r.m.s. duration of the pump beams is ${t}_{0}=\sqrt{2/3}{\sigma }_{t}=$ 6.1 ps

In order to make the conversion from critical pulse energy to *χ*^(3)^ (see below), the pulse duration is required. The FELIX laser produces trains of intense, tuneable, short pulses. The train is emitted at 10 Hz in so called macropulse bunches, which contain approximately 200 micropulses each, at a repetition rate of 25 MHz. The pulse duration can be estimated from the spectrum since the pulses are approximately bandwidth limited. For a Gaussian pulse, its r.m.s. intensity duration *t*_0_ = 1/4*πσ*_*ν*_ where the r.m.s. intensity bandwidth in frequency *ν* averaged over the macropulse is typically about *σ*_*ν*_/*ν* ≈ 0.3% corresponding to a pulse duration of a few picoseconds, and there was little variation throughout the experiments. For this work, we made use of the fact that, when off-resonance, the measured *E*_3_ as a function of the delay between the inputs **k**_1,2_ gives a third-order autocorrelation (3AC), shown in (Fig. 4). For a Gaussian pulse, the r.m.s. duration is ${t}_{0}={\sigma }_{t}\sqrt{2/3}=$ 6.1 ps, where *σ*_*t*_ is the r.m.s. duration of the 3AC given on the figure. For the data in Table 1, we took the value of *t*_0_ from the 3AC measurement of Fig. 4, and we assumed that it was constant for all experiments.

## Supplementary information

Supplementary materials

## Data Availability

The data for this work are freely available^28^.
