# Supplementary material for: Highly efficient THz four-wave mixing in doped silicon
Source: Light Sci Appl. 2021 Apr 1;10:71. doi: 10.1038/s41377-021-00509-6 (PMC8016830; doi:10.1038/s41377-021-00509-6)
Supplement: Supplementary file 1 — Supplementary materials [file 41377_2021_509_MOESM1_ESM.pdf]

Supplementary material for Dessmann *et al.* "Highly efficient THz four-wave mixing in doped silicon"

Nils Dessmann,[1,*] Nguyen H. Le,[2,†] Viktoria Eless,[1] Steven Chick,[2] Kamyar Saeedi,[1] Alberto Perez-Delgado,[2] Sergey G. Pavlov,[3] Alexander F.G. van der Meer,[1] Konstantin L. Litvinenko,[2] Ian Galbraith,[4] Nikolay V. Abrosimov,[5] Helge Riemann,[5] Carl R. Pidgeon,[4] Gabriel Aeppli,[6,7,8] Britta Redlich,[1] and Benedict N. Murdin[2]

[1] *Radboud University, Institute for Molecules and Materials, HFML-FELIX, Nijmegen, The Netherlands*
[2] *Advanced Technology Institute and Department of Physics, University of Surrey, Guildford, GU2 7XH, UK*
[3] *Institute of Optical Sensor Systems, German Aerospace Center, Berlin, Germany*
[4] *Institute of Photonics and Quantum Sciences, SUPA, Heriot-Watt University, Edinburgh, UK*
[5] *Leibniz-Institut für Kristallzüchtung (IKZ), Berlin, Germany*
[6] *Laboratory for Solid State Physics, ETH Zürich, Zürich, CH-8093, Switzerland*
[7] *Institut de Physique, EPFL, Lausanne, CH-1015, Switzerland*
[8] *Paul Scherrer Institute, Villigen, CH-5232, Switzerland*

In these Supplementary Materials we provide some additional experimental details about the spectral dependence of the signal obtained in the experiment in section I. We provide details of the calculation of $\chi^{(3)}$ for a two level model in section II. In section III this two-level result is extended to include Gaussian inhomogeneous broadening and produce the approximate results for the non-linear susceptibility in Eq. (4) of the main manuscript. Finally, section IV provides the conversion of the experimental critical energy from Fig 2 of the main manuscript into values of $\chi^{(3)}$ as given in Table I of the main manuscript, assuming Gaussian pulses and a Gaussian beam profile (i.e. we derive the equation given in the last section of the Methods section of the main manuscript for $E_c(\chi^{(3)})$ and the calculation of the factor f which appears therein).

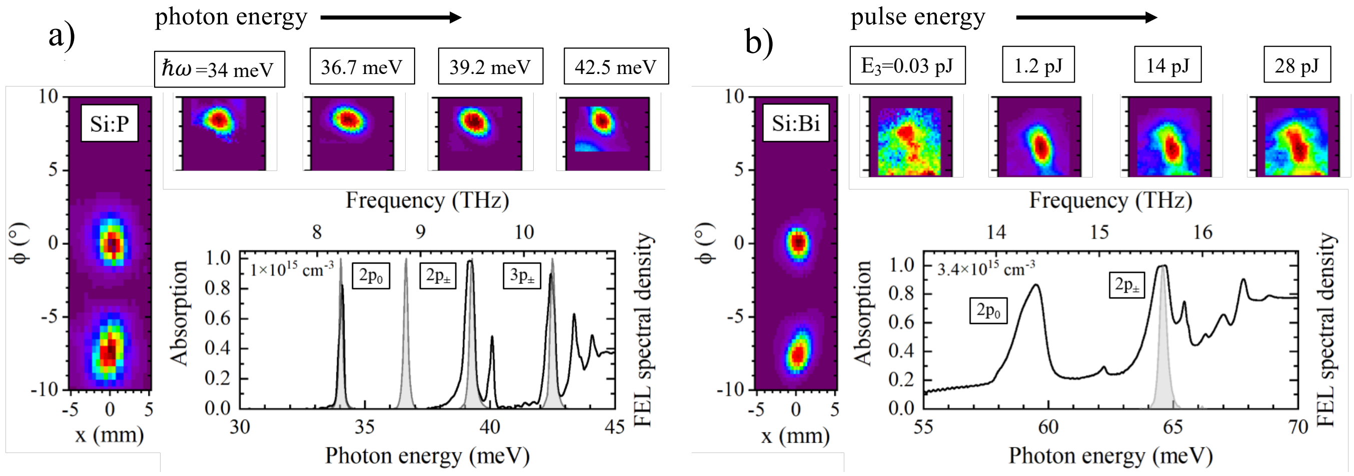

FIG. S1. DFWM beam profiles taken with a far-field scanning iris for various Si:P (a) and Si:Bi (b) transitions and intensities. The FTIR absorption spectra are shown with black lines and the grey shaded curves indicate the laser spectrum used. Absorption is defined as $1 - I_{\mathrm{out}}/I_{\mathrm{bckgnd}}$ where I_{out} is the transmitted intensity normalised by the system response, and I_{bckgnd} is the value of I_{out} between resonances. In (a) there are four different laser frequencies indicated ($\hbar\omega$)and four corresponding beam profiles. In (b) a single laser frequency is used an the beam profiles are shown for four different laser intensities indicated by the resulting output pulse energies (E_3). Noise caused by scatter from the pumps is evident when the efficiency is low, at low intensity and also at high intensity when the DFWM saturates (while scatter does not). The color scale for each beam image has been normalized.

* corresponding author, email:nils.dessmann@ru.nl
† corresponding author, email:h.le@surrey.ac.uk

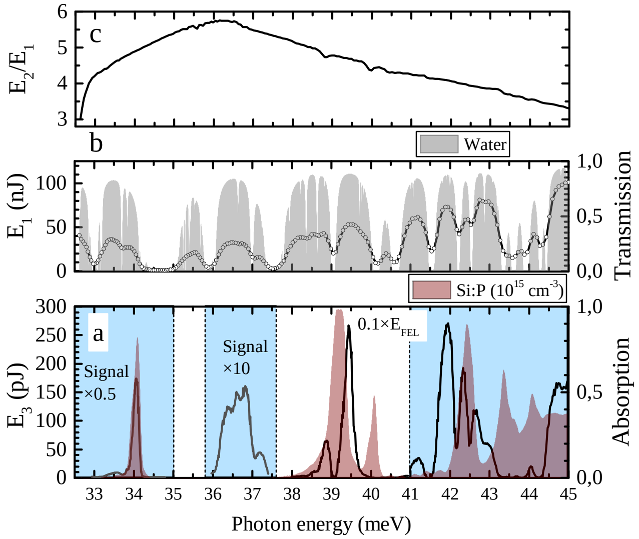

FIG. S2. Laser frequency dependence of the output. (a) The ratio of energies of the input pulses, $x = E_2/E_1$, which was set to approximately 4 by choice of beam-splitter and attenuators, and monitored thoughout. (b) The modelled spectrum for air transmission is shown in grey (right axis). The measured pulse energy for beam 1 (left axis) measured with a power meter before the sample, and corrected for the cryostat window and the sample surface reflection. The typical laser spectral r.m.s. width was $\sigma_f/f = 0.3\%$, i.e. 0.1 meV as shown in Fig. S1. (c) The FTIR absorption spectrum of the $n_D = 10^{16}$ cm^{-3} Si:P sample is shown in red (right axis). The thick black curve is the DFWM output for this sample (left axis), which follows notionally the product of the FTIR and air transmission from (b). For clarity two portions of the DFWM signal have been scaled by factors of 0.5 and 10 as indicated, and in one section of the scan the laser input beams were attenuated by a factor of 0.1 as indicated.

I. SPECTRA OF THE SMALL SIGNAL ABSORPTION, THE DFWM SIGNAL AND THE LASER

We monitored the DFWM output beam throughout the experiment in order to ensure that it was clearly identifiable and separated from scatter from the pump beams. Example beam images are shown in Fig. S1, which also shows the laser spectrum and sample absorption spectrum for comparison.

An example calibration of the ratio between E_2 and E_1 and the absolute pulse energy E_1 as a function of laser frequency is given in Fig. S2. The figure also shows the transmission of air, and the resulting laser frequency dependence of the output pulse energy, which clearly scales with the energy of the pump pulse and the absorption cross-section, as expected.

II. TWO-LEVEL MODEL FOR THIRD ORDER SUSCEPTIBILITY

The Hamiltonian for the interaction of light with the donor is

$$H = H_0 + V(t), \tag{1}$$

where H_0 is the Hamiltonian of the donor's electron with eigenstates $H_0 |j\rangle = \hbar\omega_j |j\rangle$ and $V(t) = -\mu F(t)$ is the dipole potential with electric field $F(t)$.

The density matrix of the donor's electron evolves according to the quantum Liouville equation

$$\frac{d\rho}{dt} = -\frac{i}{\hbar}[H_0 + V(t), \rho] + W, \tag{2}$$

where W is responsible for relaxation and dephasing processes. In the basis of unperturbed states $|j\rangle$ we take

$$W_{jk} = \langle j| W |k\rangle = -\Gamma_{jk}(\rho_{jk} - \rho_{jk}^{(\mathrm{eq})}), \tag{3}$$

where Γ_{jk} is the damping rate of $\rho_{jk} \equiv \langle j| \rho |k\rangle$ back to its value at thermal equilibrium in the dark, $\rho_{jk}^{(\mathrm{eq})}$. The rate Γ_{jj} is the population relaxation rate of the jth level and the $\Gamma_{j,k}$, with $j \neq k$, are the dephasing rates of the

off-diagonal elements. At low temperature we can assume that $\rho_{gg}^{(\mathrm{eq})} = 1$ while the equilibrium values of the other elements are zero.

From Eq. (2) we have

$$\frac{d\rho_{jk}}{dt} = -\frac{i}{\hbar}\left(\langle j|\,[H_0,\rho]\,|k\rangle + \langle j|\,[V(t),\rho]\,|k\rangle\right) - \Gamma_{jk}(\rho_{jk} - \rho_{jk}^{(\mathrm{eq})}). \tag{4}$$

Since $\langle j|\,[H_0,\rho]\,|k\rangle = \hbar\omega_{jk}\rho_{jk}$ with $\omega_{jk} = \omega_j - \omega_k$, and

$$\langle j|\,[V(t),\rho]\,|k\rangle = -F(t)\,[\langle j|\,\mu\rho\,|k\rangle - \langle j|\,\rho\mu\,|k\rangle] = -F(t)\sum_l [\mu_{jl}\rho_{lk} - \rho_{jl}\mu_{lk}],$$

where the last step follows from inserting $|l\rangle\langle l| = \mathbb{1}$ between the operators μ and ρ, we have

$$\frac{d\rho_{jk}}{dt} + (i\omega_{jk} + \Gamma_{jk})\rho_{jk} = \frac{i}{\hbar}F(t)\sum_l [\mu_{jl}\rho_{lk} - \rho_{jl}\mu_{lk}] + \Gamma_{jk}\rho_{jk}^{(\mathrm{eq})}. \tag{5}$$

One can express the solution in terms of the Green function which is given by

$$\frac{dG_{jk}}{dt} + (i\omega_{jk} + \Gamma_{jk})G_{jk} = \delta(t), \tag{6}$$

where $\delta(t)$ is the Dirac delta function, which yields

$$G_{jk}(t) = \mathcal{G}_{jk}(t)e^{-i\omega_{jk}t}, \tag{7}$$

where

$$\mathcal{G}_{jk}(t) = \Theta(t)e^{-\Gamma_{jk}t}, \tag{8}$$

and $\Theta(t)$ is the step function. The function $\mathcal{G}_{jk}(t)$ can be thought of as the envelope of the Green function. One can verify that $G_{jk}(t)$ is the solution of Eq. (6) by using $d\Theta(t)/dt = \delta(t)$. For $jk \neq gg$, the equilibrium value is zero at low temperature, hence

$$\rho_{jk}(t) = \frac{i}{\hbar}\int_{-\infty}^{\infty} dt' G_{jk}(t-t')F(t')\sum_l [\mu_{jl}\rho_{lk}(t') - \rho_{jl}(t')\mu_{lk}], \tag{9}$$

where we have assumed the initial condition $\rho_{jk}(-\infty) = 0$ for $jk \neq gg$. Although this integral equation does not provide a direct evaluation of $\rho_{jk}(t)$ since $\rho_{lk}(t')$ on the RHS is unknown, it is useful for obtaining the perturbative solution for weak field. We expand $\rho_{jk}(t) = \sum_n \rho_{jk}^{(n)}(t)$, then Eq. (9) gives

$$\rho_{jk}^{(n)}(t) \approx \frac{i}{\hbar}\int_{-\infty}^{\infty} dt' G_{jk}(t-t')F(t')\sum_l [\mu_{jl}\rho_{lk}^{(n-1)}(t') - \rho_{jl}^{(n-1)}(t')\mu_{lk}], \tag{10}$$

which can be applied successively to find ρ_{jk} up to the nth order [1].

When the carrier frequency of the pulse is tuned close to the transition frequency between the ground state $|g\rangle$ and excited state $|e\rangle$, one can use the *two level approximation*. Now there are only two damping rates: The population relaxation rate, $\Gamma_{ee} = 1/T_1$ and the dephasing rate, $\Gamma_{eg} = \Gamma_{ge} = 1/T_2$. The time-dependent polarization of a sample with donor density n_D is

$$P(t) = n_D\,\mathrm{Tr}[\mu\rho(t)] = n_D[\mu_{ge}\rho_{eg}(t) + \mu_{eg}\rho_{ge}(t)] = n_D[\mu_{ge}\rho_{eg}(t) + \mathrm{c.c}], \tag{11}$$

so all we need to find is $\rho_{eg}(t)$. The zero order elements of $\rho(t)$ are $\rho_{gg}^{(0)} = 1$ and $\rho_{jk\neq gg}^{(0)} = 0$. Substituting this into Eq. (10) and using $\mu_{gg} = \mu_{ee} = 0$, one obtain the first order off-diagonal elements

$$\begin{aligned} \rho_{eg}^{(1)}(t) &= i\frac{\mu_{eg}}{\hbar}\int_{-\infty}^{\infty} dt_1 G_{eg}(t-t_1)F(t_1), \\ \rho_{ge}^{(1)}(t) &= -i\frac{\mu_{ge}}{\hbar}\int_{-\infty}^{\infty} dt_1 G_{ge}(t-t_1)F(t_1), \end{aligned} \tag{12}$$

and then the second order population

$$\rho_{ee}^{(2)}(t) = \frac{i}{\hbar}\int_{-\infty}^{\infty} dt_2 G_{ee}(t-t_2)F(t_2)\left[\mu_{eg}\rho_{ge}^{(1)}(t_2) - \rho_{eg}^{(1)}(t_2)\mu_{ge}\right]$$
$$= -\frac{|\mu_{eg}|^2}{\hbar^2}\int_{-\infty}^{\infty} dt_2 G_{ee}(t-t_2)F(t_2)\int_{-\infty}^{\infty} dt_1[G_{eg}(t_2-t_1)+G_{ge}(t_2-t_1)]F(t_1), \tag{13}$$

and finally the third order off diagonal element

$$\rho_{eg}^{(3)}(t) = i\frac{\mu_{eg}}{\hbar}\int_{-\infty}^{\infty} dt_3 G_{eg}(t-t_3)F(t_3)(\rho_{gg}^{(2)}(t_3)-\rho_{ee}^{(2)}(t_3)) = -2i\frac{\mu_{eg}}{\hbar}\int_{-\infty}^{\infty} dt_3 G_{eg}(t-t_3)F(t_3)\rho_{ee}^{(2)}(t_3), \tag{14}$$

where the last step follows from $\rho_{gg}^{(2)}(t)+\rho_{ee}^{(2)}(t)=0$ as the total population is conserved, hence

$$\rho_{eg}^{(3)}(t) = -2i\frac{\mu_{eg}|\mu_{eg}|^2}{\hbar^3}\int_{-\infty}^{\infty} dt_3 G_{eg}(t-t_3)F(t_3)\int_{-\infty}^{\infty} dt_2 G_{ee}(t_3-t_2)F(t_2)\int_{-\infty}^{\infty} dt_1[G_{eg}(t_2-t_1)+G_{ge}(t_2-t_1)]F(t_1). \tag{15}$$

The polarization up to the third order is then $P(t)=P^{(1)}(t)+P^{(3)}(t)$ where

$$P^{(1)}(t) = n_D[\mu_{ge}\rho_{eg}^{(1)}(t)+\text{c.c}],$$
$$P^{(3)}(t) = n_D[\mu_{ge}\rho_{eg}^{(3)}(t)+\text{c.c}]. \tag{16}$$

Note that the second order term $P^{(2)}(t)=0$ since $\rho_{eg}^{(2)}(t)=0$.

We write the electric fields of the pump and rephasing pulses as

$$F_1(z,t) = \frac{1}{2}\mathcal{F}_1(z,t)e^{i(\mathbf{k}_1.\mathbf{r}-\omega_0 t)}+\text{c.c},$$
$$F_2(z,t) = \frac{1}{2}\mathcal{F}_2(z,t)e^{i(\mathbf{k}_2.\mathbf{r}-\omega_0 t)}+\text{c.c}, \tag{17}$$

where ω_0 is the carrier frequency, $\mathcal{F}_{1,2}(z,t)$ the pulse envelopes, and the z axis is parallel to the light beams and hence perpendicular to the sample surface. The total electric field is

$$F(z,t) = F_1(z,t)+F_2(z,t) = \frac{1}{2}\mathcal{F}_1(z,t)e^{i(\boldsymbol{k}_1.\boldsymbol{r}-\omega_0 t)}+\text{c.c}+\frac{1}{2}\mathcal{F}_2(z,t)e^{i(\boldsymbol{k}_2.\boldsymbol{r}-\omega_0 t)}+\text{c.c}, \tag{18}$$

To find the 3rd-order polarization we substitute the electric field of Eq. (18) into Eq. (15). Each electric field is a sum of 4 terms, and an expansion yields $4^3=64$ terms in $\rho_{eg}^{(3)}(t)$. However, we need to keep only the terms that propagate in the $2\boldsymbol{k}_2-\boldsymbol{k}_1$ direction of the ouput field, and we can also neglect the counter rotating terms. Upon inspection there are only two terms left, and the third order polarization is

$$P^{(3)}(z,t) = \frac{1}{2}\mathcal{P}^{(3)}(z,t)e^{i(kz-\omega_0 t)}+c.c, \tag{19}$$

where the envelope is

$$\mathcal{P}^{(3)}(z,t) = -\frac{i\,n_D|\mu_{eg}|^2}{\hbar^3}\frac{\beta_1(z,t)+\beta_2(z,t)}{2},$$
$$\beta_1(z,t) = e^{i\Delta t}\int_{-\infty}^{t} dt_3 e^{-(t-t_3)/T_2}\tilde{\mathcal{F}}_2(z,t_3)\int_{-\infty}^{t_3} dt_2 e^{-(t_3-t_2)/T_1}\tilde{\mathcal{F}}_2(z,t_2)\int_{-\infty}^{t_2} dt_1 e^{-(t_2-t_1)/T_2}\tilde{\mathcal{F}}_1^*(z,t_1),$$
$$\beta_2(z,t) = e^{i\Delta t}\int_{-\infty}^{t} dt_3 e^{-(t-t_3)/T_2}\tilde{\mathcal{F}}_2(z,t_3)\int_{-\infty}^{t_3} dt_2 e^{-(t_3-t_2)/T_1}\tilde{\mathcal{F}}_1^*(t_2)\int_{-\infty}^{t_2} dt_1 e^{-(t_2-t_1)/T_2}\tilde{\mathcal{F}}_2(z,t_1), \tag{20}$$

where $\Delta=\omega_0-\omega_{eg}$ is the detuning and $\tilde{\mathcal{F}}_{1,2}(z,t)=\mathcal{F}_{1,2}(z,t)e^{-i\Delta t}$.

In the monochromatic limit of infinitely long pulses, and when there is no loss due to absorption, $\mathcal{F}_{1,2}(z,t)$ and hence $\mathcal{P}^{(3)}(z,t)$ are constants. It's now straightforward to carry out the integrals in Eq. (20), which yields

$$\mathcal{P}^{(3)} = \epsilon_0\chi^{(3)}\mathcal{F}_1^*\mathcal{F}_2^2, \tag{21}$$

where the third order susceptibility is

$$\chi^{(3)} = \frac{n_D|\mu_{eg}|^4}{\epsilon_0\hbar^3}\frac{T_1T_2^2}{(1+\Delta^2T_2^2)(i+\Delta T_2)}. \tag{22}$$

On resonance $\Delta \ll 1/T_2$ and

$$\chi^{(3)} \approx -i\frac{n_D|\mu_{eg}|^4}{\epsilon_0\hbar^3}T_1T_2^2. \tag{23}$$

while sufficiently far from resonance $\Delta \gg 1/T_2$ and in this case

$$\chi^{(3)} \approx \frac{n_D|\mu_{eg}|^4}{\epsilon_0\hbar^3}\frac{T_1}{\Delta^3T_2}. \tag{24}$$

III. EFFECT OF INHOMOGENEITY IN THE TWO LEVEL MODEL FOR THIRD ORDER SUSCEPTIBILITY

For an inhomogeneously broadened sample we model the distribution of the transition frequency, ω'_{eg}, by a Gaussian

$$g(\omega'_{eg}-\omega_{eg}) = \frac{1}{\sqrt{2\pi}\Gamma}\exp\left[-\frac{\left(\omega'_{eg}-\omega_{eg}\right)^2}{2\Gamma^2}\right], \tag{25}$$

where $\sqrt{2\ln(2)}\Gamma = 1/T_{\rm inh}$ is the half width at half maximum of the distribution in angular frequency, which defines $T_{\rm inh}$, the inhomogeneous contribution to the dephasing time, and ω_{eg} the peak frequency of the broadened transition. For such a sample the third order susceptibility given in Eq. (22) has to be averaged over the distribution of ω'_{eg}:

$$\chi^{(3)} = \frac{n_D|\mu_{eg}|^4}{\epsilon_0\hbar^3}T_1T_2^2\eta, \tag{26}$$

where

$$\eta = \int_{-\infty}^{\infty} d\delta\,\frac{g(\delta)}{(1+(\Delta-\delta)^2T_2^2)(i+(\Delta-\delta)T_2)}, \tag{27}$$

and $\delta = \omega'_{eg}-\omega_{eg}$. $\Delta = \omega_0-\omega_{eg}$ as before, which now means the detuning from the centre of mass. If $\Gamma T_2 \ll 1$ then we have a homogeneous line, and $g(\delta)$ is only large near $\delta = 0$, and since g has unit area $\eta \approx 1/(1+\Delta^2T_2^2)(i+\Delta T_2)$ and we recover Eq. (22).

Far from resonance, $\Delta \gg 1/T_2$ and $g(\delta)$ is only large when δ is small compared with Δ so

$$\eta \approx \frac{1}{\Delta^3T_2^3}\int_{-\infty}^{\infty} d\delta\, g(\delta) = \frac{1}{\Delta^3T_2^3}, \tag{28}$$

and we recover Eq (24), which evidently holds for both homogeneously and inhomogeneously broadened transitions.

When the laser is on resonance $\Delta \ll \Gamma$,

$$\begin{aligned}\eta &\approx \int_{-\infty}^{\infty} d\delta\,\frac{g(\delta)}{(1+\delta^2T_2^2)(i-\delta T_2)} = -i\int_{-\infty}^{\infty} d\delta\,\frac{g(\delta)}{(1+\delta^2T_2^2)^2} \\ &= \frac{-i}{2(T_2\Gamma)^2} - i\sqrt{\pi}\frac{(\Gamma T_2)^2-1}{2\sqrt{2}(\Gamma T_2)^3}e^{1/2(\Gamma T_2)^2}\mathrm{Erfc}(1/\sqrt{2}\Gamma T_2)\end{aligned} \tag{29}$$

where Erfc is the complementary error function and on the first line we used the fact that the real part of the integrand is odd. For a homogeneously broadened line $T_{\rm inh} \gg T_2$ so $\Gamma T_2 \ll 1$ and

$$\eta \approx -i, \tag{30}$$

(as it must be comparing Eqs (23) and (26)). For an inhomogenously broadened line $T_{\rm inh} \ll T_2$, so $\Gamma T_2 \gg 1$ and $e^{1/2(\Gamma T_2)^2}[1-\mathrm{Erf}(1/\sqrt{2}\Gamma T_2)] \approx 1$,

$$\eta \approx -i\frac{\sqrt{\pi}}{2\sqrt{2}\Gamma T_2} = -i\frac{\sqrt{\pi\ln 2}}{2}\frac{T_{\rm inh}}{T_2}. \tag{31}$$

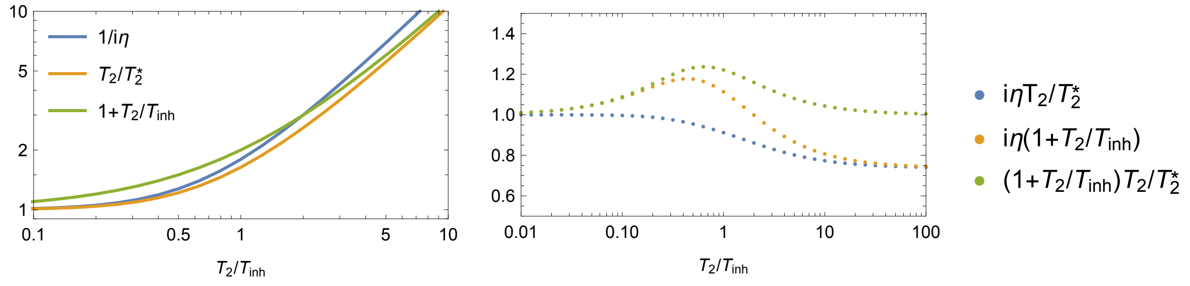

FIG. S3. A comparison of the terms in the approximations of Eq.s (34) & (36). The left panel shows the results of Eq. (29) (blue line) and Eq. (33) (yellow) and the right hand side of Eq. (34) times T_2 (green). The right panel shows ratios of the three curves from the left panel, showing the agreement for a wide range of $T_2/T_{\rm inh}$ (homogeneous broadening means small $T_2/T_{\rm inh}$ and inhomogeneous broadening means large $T_2/T_{\rm inh}$).

The linear absorption line shape in the presence of both homogeneous and inhomogeneous broadening is given by a Voigt profile (Main Text Ref [17]) which is the convolution of the Lorentzian of width $1/T_2$ in angular frequency for the homogeneous contribution, and the Gaussian $g(\delta)$ inhomogeneous contribution:

$$V(x) = \frac{1}{\pi}\int_{-\infty}^{\infty} T_2 d\delta \, \frac{g(\delta)}{(1+(x-T_2\delta)^2)}, \tag{32}$$

where $x = T_2\Delta$. The half width of the Voigt lineshape in angular frequency, $1/T_2^*$, is given by

$$\frac{T_2}{T_2^*} = V^{-1}\left(V(0)/2\right). \tag{33}$$

T_2^* must be found numerically in general, though the asymptotic limits are $T_2^* \approx T_{\rm inh}$ for $T_{\rm inh} \ll T_2$ and $T_2^* \approx T_2$ for $T_{\rm inh} \gg T_2$, and a common analytical approximation based on these limits is (Main Text Ref [17])

$$\frac{1}{T_2^*} \approx \frac{1}{T_2} + \frac{1}{T_{\rm inh}}. \tag{34}$$

Similarly, a useful analytical approximation for η based on its asymptotic behaviour Eq.s (30) & (31) is

$$\frac{1}{i\eta} \approx 1 + \frac{2}{\sqrt{\pi \ln 2}}\frac{T_2}{T_{\rm inh}}. \tag{35}$$

Using $\frac{2}{\sqrt{\pi \ln 2}} \sim 1$ in Eq. (35) and substituting (34), or by inspection of Fig S3, a further simplifying approximation for η is

$$\eta \approx -i\frac{T_2^*}{T_2}. \tag{36}$$

These approximations are shown on Fig S3, and it may be seen that they are satisfactory over a wide range of $T_2/T_{\rm inh}$. The maximum discrepancy in Eq. (36) (blue dotted line on Fig S3, when $T_2 \gg T_{\rm inh}$) is no worse than the maximum discrepancy in Eq. (34) (green dotted line on Fig S3, when $T_2 \sim T_{\rm inh}$). The yellow dotted line on Fig S3 shows the combined approximation $1/i\eta \approx 1 + T_2/T_{\rm inh}$ is also satisfactory. Substituting Eq. (36) into Eq. (26) gives

$$\chi^{(3)} \approx -i\frac{n_D|\mu_{eg}|^4}{\epsilon_0\hbar^3}T_1T_2T_2^*. \tag{37}$$

This approximation now covers the resonant situation for both homogeneous and inhomogenous broadening, as did Eq (24) for the off-resonant situation, given in the Main Text as Eq. (4). For self-consistency within Main Text we use the approximate values resulting from Eq. (37) in Table I.

IV. EFFECT OF FINITE PULSES AND LOSS IN THE TWO LEVEL MODEL FOR THIRD ORDER SUSCEPTIBILITY

A. Wave propagation

To get the output field from the nonlinear polarisation we need to solve the wave propagation equation. The electric field satisfies the Maxwell equation [2]

$$\nabla^2 F - \frac{1}{c^2}\frac{\partial^2 F}{\partial t^2} = \mu_0 \frac{\partial^2 P_{\text{tot}}}{\partial t^2}. \tag{38}$$

The total polarization in a doped semiconductor is the sum of the host's polarization (silicon in our case), $P_{\text{host}} = \epsilon_0(\epsilon_r - 1)E \approx \epsilon_0(n^2-1)F$ where n is the refractive index of the host, and the polarization component P of the donors that propagates in the $\boldsymbol{k} = 2\boldsymbol{k}_2 - \boldsymbol{k}_1$ direction. Substituting $P_{\text{tot}} = \epsilon_0(n^2-1)F + P$ yields

$$\nabla^2 F - \frac{n^2}{c^2}\frac{\partial^2 F}{\partial t^2} = \mu_0 \frac{\partial^2 P}{\partial t^2}. \tag{39}$$

For general non-monochromatic pulses with a finite frequency broadening this equation is best solved with Fourier transform. Starting with

$$\begin{aligned} F(z,t) &= \frac{1}{2}\mathcal{F}(z,t)e^{i(\mathbf{k}.\mathbf{r}-\omega_0 t)} + c.c., \\ P(z,t) &= \frac{1}{2}\mathcal{P}(z,t)e^{i(\mathbf{k}.\mathbf{r}-\omega_0 t)} + c.c., \end{aligned} \tag{40}$$

where the envelope functions have the Fourier transforms

$$\begin{aligned} \mathcal{F}(z,t) &= \int_{-\infty}^{\infty} d\omega \hat{\mathcal{F}}(z,\omega)e^{-i\omega t}, \\ \mathcal{P}(z,t) &= \int_{-\infty}^{\infty} d\omega \hat{\mathcal{P}}(z,\omega)e^{-i\omega t}, \end{aligned} \tag{41}$$

where ω is the deviation from ω_0. Substituting Eqs. (41) and (40) into Eq. (39) we obtain the propagation equation for each Fourier component

$$\left(\frac{\partial^2}{\partial z^2} + 2ik\frac{\partial}{\partial z} - k^2\right)\hat{\mathcal{F}}(z,\omega) + \frac{(\omega+\omega_0)^2 n^2}{c^2}\hat{\mathcal{F}}(z,\omega) = -(\omega+\omega_0)^2\mu_0\hat{\mathcal{P}}(z,\omega). \tag{42}$$

When the first spatial derivative of the envelope functions changes very little over a wavelength, which is true for our experiment because the wavelength is of the order of 0.01 mm while the length of the Gaussian pulse is of the order of 2 mm, we have

$$\left|\frac{1}{k}\frac{\partial^2 \hat{\mathcal{F}}(z,\omega)}{\partial z^2}\right| \ll \left|\frac{\partial \hat{\mathcal{F}}(z,\omega)}{\partial z}\right|. \tag{43}$$

Neglecting the 2nd order spatial derivative in Eq. (42) and using $k = \omega n/c$ and $\mu_0 = 1/(\epsilon_0 c^2)$, we obtain

$$\frac{\partial}{\partial z}\hat{\mathcal{F}}(z,\omega) = \frac{i(\omega+\omega_0)\mathcal{Z}}{2}\hat{\mathcal{P}}(z,\omega), \tag{44}$$

where $\mathcal{Z} = \mathcal{Z}_0/n$ and $\mathcal{Z}_0 = 1/c\epsilon_0$ is the impedance of the vacuum. In our experiment the frequency bandwidth is much smaller than the carrier frequency, $\omega \ll \omega_0$, hence $\omega + \omega_0 \approx \omega_0$ and

$$\frac{\partial}{\partial z}\hat{\mathcal{F}}(z,\omega) \approx \frac{i\omega_0\mathcal{Z}}{2}\hat{\mathcal{P}}(z,\omega). \tag{45}$$

In the monochromatic limit of infinitely long pulses there is only a single mode, $\omega = 0$, in the spectrum. Assuming that there is no loss, substituting the third order polarisation from Eq. (21) and integrating, we obtain the four wave mixing output field

$$\mathcal{F}_3^{(\text{out})} = \frac{i\omega_0\epsilon_0\mathcal{Z}\chi^{(3)}L}{2}\mathcal{F}_1^*\mathcal{F}_2^2, \tag{46}$$

where L is the thickness of the sample.

B. Loss of the input due to absorption

In our experiment the pump and the rephasing pulses are approximately Gaussian before entering the sample

$$\mathcal{F}_{1,2}(z,t) = \mathcal{F}_{1,2}(0,0)\mathcal{S}(z,t), \tag{47}$$

where the time envelope at the beginning of the sample, $z = 0$, is

$$\mathcal{S}(0,t) = e^{-t^2/2\tau^2}, \tag{48}$$

where τ is the r.m.s. duration of the electric field, and is related to the r.m.s duration of the intensity in the Main Text, t_0, by $\tau = \sqrt{2}t_0$.

As the input pulses travel into the sample they are attenuated by loss due to absorption. This is predominantly a linear optical process due to the 1st order polarisation, which is

$$P^{(1)}(z,t) = \frac{1}{2}\mathcal{P}^{(1)}(z,t)e^{i(\boldsymbol{k}_{\rm in}.\boldsymbol{r}-\omega t)} + c.c., \tag{49}$$

where $\boldsymbol{k}_{\rm in}$ is the wave vector of the input field, either the pump or rephasing. Substituting the electric field from Eq. (17) into Eq. (12) and then the resulting into Eq. (16), we obtain

$$\mathcal{P}^{(1)}(z,t) = i\,n_D\frac{|\mu_{eg}|^2}{\hbar}e^{i\Delta t}\int_{-\infty}^{t} dt_1 e^{-(t-t_1)/T_2}\mathcal{F}(z,t_1)e^{-i\Delta t_1}. \tag{50}$$

The input field inside the sample is given by the wave propagation equation, Eq. (45), which can be solved exactly by first taking the Fourier transform

$$\mathcal{F}(z,t) = \int_{-\infty}^{\infty} d\omega\hat{\mathcal{F}}(z,\omega)e^{-i\omega t}, \tag{51}$$

then

$$\begin{aligned}\mathcal{P}^{(1)}(z,t) &= i\,n_D\frac{|\mu_{eg}|^2}{\hbar}e^{i\Delta t}\int_{-\infty}^{t} dt_1 e^{-(t-t_1)/T_2}e^{-i\Delta t_1}\int_{-\infty}^{\infty} d\omega\hat{\mathcal{F}}(z,\omega)e^{-i\omega t_1}\\ &= i\,n_D\frac{|\mu_{eg}|^2}{\hbar}\int_{-\infty}^{\infty} d\omega\frac{\hat{\mathcal{F}}(z,\omega)}{1/T_2 - i(\Delta+\omega)}e^{-i\omega t},\end{aligned} \tag{52}$$

so

$$\hat{\mathcal{P}}^{(1)}(\omega,t) = i\,n_D\frac{|\mu_{eg}|^2}{\hbar}\frac{\hat{\mathcal{F}}(z,\omega)}{1/T_2 - i(\Delta+\omega)}. \tag{53}$$

Substituting Eq.(53) into Eq. (45) we obtain

$$\frac{\partial\hat{\mathcal{F}}(z,\omega)}{\partial z} = -\alpha(\Delta,\omega)\hat{\mathcal{F}}(z,\omega), \tag{54}$$

which is the usual equation for absorption in the frequency domain where the frequency dependent absorption coefficient is

$$\alpha(\Delta,\omega) = \frac{\omega_0\mathcal{Z}n_D|\mu_{eg}|^2}{2\hbar}\frac{1}{1/T_2 - i(\Delta+\omega)}. \tag{55}$$

The Fourier component of the input pulses inside the sample is thus given by a simple exponential

$$\hat{\mathcal{F}}(z,\omega) = e^{-\alpha(\Delta,\omega)z}\hat{\mathcal{F}}(0,\omega). \tag{56}$$

Therefore, the time envelope of the field inside the sample has the form

$$\mathcal{S}(z,t) = \int_{-\infty}^{\infty} d\omega\,\hat{\mathcal{S}}(z,\omega)e^{-i\omega t}, \tag{57}$$

where the Fourier component is

$$\hat{\mathcal{S}}(z,\omega) = e^{-\alpha(\Delta,\omega)z}\hat{\mathcal{S}}(0,\omega), \tag{58}$$

and

$$\hat{\mathcal{S}}(0,\omega) = \frac{1}{2\pi}\int_{-\infty}^{\infty} dt\,S(0,t)e^{i\omega t} = \frac{\tau}{\sqrt{2\pi}}e^{-\omega^2\tau^2/2}. \tag{59}$$

C. Output field

Substituting Eq. (47) into Eq. (20) the third order polarisation is now

$$\mathcal{P}^{(3)}(z,t) = -i\frac{n_D|\mu_{eg}|^4}{\hbar^3}\mathcal{F}_1^*(0,0)\mathcal{F}_2^2(0,0)\frac{Q_1(\Delta,z,t)+Q_2(\Delta,z,t)}{2}, \tag{60}$$

where

$$\mathcal{Q}_1(\Delta,z,t) = e^{i\Delta t}\int_{-\infty}^{t} dt_3 e^{-(t-t_3)/T_2}\tilde{\mathcal{S}}(\Delta,z,t_3)\int_{-\infty}^{t_3} dt_2 e^{-(t_3-t_2)/T_1}\tilde{\mathcal{S}}(\Delta,z,t_2)\int_{-\infty}^{t_2} dt_1 e^{-(t_2-t_1)/T_2}\tilde{\mathcal{S}}^*(\Delta,z,t_1),$$
$$\mathcal{Q}_2(\Delta,z,t) = e^{i\Delta t}\int_{-\infty}^{t} dt_3 e^{-(t-t_3)/T_2}\tilde{\mathcal{S}}(\Delta,z,t_3)\int_{-\infty}^{t_3} dt_2 e^{-(t_3-t_2)/T_1}\tilde{\mathcal{S}}^*(\Delta,z,t_2)\int_{-\infty}^{t_2} dt_1 e^{-(t_2-t_1)/T_2}\tilde{\mathcal{S}}(\Delta,z,t_1), \tag{61}$$

where $\tilde{\mathcal{S}}(\Delta,z,t) = \mathcal{S}(z,t)e^{-i\Delta t}$. We now use the Fourier transform of $S(z,t)$ in the integral of $\mathcal{Q}_1(\Delta,z,t)$

$$\int_{-\infty}^{t_2} dt_1 e^{-(t_2-t_1)/T_2} e^{i\Delta t_1}\int_{-\infty}^{\infty} d\omega_1 \mathcal{S}^*(z,\omega_1)e^{i\omega_1 t_1} = \int_{-\infty}^{\infty} d\omega_1 \mathcal{S}^*(z,\omega_1)\frac{e^{i(\Delta+\omega_1)t_2}}{1/T_2 + i(\Delta+\omega_1)},$$

and

$$\int_{-\infty}^{t_3} dt_2\, e^{-(t_3-t_2)/T_1}e^{-i\Delta t_2}e^{i(\Delta+\omega_1)t_2}e^{-i\omega_2 t_2} = \frac{e^{i(\omega_1-\omega_2)t_3}}{1/T_1 + i(\omega_1-\omega_2)},$$

and

$$\int_{-\infty}^{t} dt_3\, e^{-(t-t_3)/T_2}e^{-i\Delta t_3}e^{i(\omega_1-\omega_2-\omega_3)t_3} = \frac{e^{-i(\Delta-\omega_1+\omega_2+\omega_3)t}}{1/T_2 - i(\Delta-\omega_1+\omega_2+\omega_3)}.$$

So

$$Q_1(\Delta,z,t) = \int_{-\infty}^{\infty} d\omega_3\int_{-\infty}^{\infty} d\omega_2\int_{-\infty}^{\infty} d\omega_1 \frac{e^{i(\omega_1-\omega_2-\omega_3)t}\hat{\mathcal{S}}(z,\omega_3)\hat{\mathcal{S}}(z,\omega_2)\hat{\mathcal{S}}^*(z,\omega_1)}{[1/T_2 - i(\Delta-\omega_1+\omega_2+\omega_3)][1/T_1 + i(\omega_1-\omega_2)][1/T_2 + i(\Delta+\omega_1)]}. \tag{62}$$

Similarly,

$$Q_2(\Delta,z,t) = \int_{-\infty}^{\infty} d\omega_3\int_{-\infty}^{\infty} d\omega_2\int_{-\infty}^{\infty} d\omega_1 \frac{e^{i(-\omega_1+\omega_2-\omega_3)t}\hat{\mathcal{S}}(z,\omega_3)\hat{\mathcal{S}}^*(z,\omega_2)\hat{\mathcal{S}}(z,\omega_1)}{[1/T_2 - i(\Delta+\omega_1-\omega_2+\omega_3)][1/T_1 - i(\omega_1-\omega_2)][1/T_2 - i(\Delta+\omega_1)]}. \tag{63}$$

The Fourier transform of $\mathcal{Q}_1(\Delta,z,t)$ is

$$\hat{\mathcal{Q}}_1(\Delta,z,\omega) = \frac{1}{2\pi}\int_{-\infty}^{\infty} dt\, \mathcal{Q}_1(\Delta,z,t)e^{i\omega t}. \tag{64}$$

Using the Dirac delta function's identity

$$\frac{1}{2\pi}\int_{-\infty}^{\infty} dt\, e^{i(\omega+\omega_1-\omega_2-\omega_3)t} = \delta(\omega+\omega_1-\omega_2-\omega_3), \tag{65}$$

we obtain

$$\hat{\mathcal{Q}}_1(\Delta,z,\omega) = \int_{-\infty}^{\infty} d\omega_2\int_{-\infty}^{\infty} d\omega_1 \frac{\hat{\mathcal{S}}(z,\omega+\omega_1-\omega_2)\hat{\mathcal{S}}(z,\omega_2)\hat{\mathcal{S}}^*(z,\omega_1)}{[1/T_2 - i(\Delta+\omega)][1/T_1 + i(\omega_1-\omega_2)][1/T_2 + i(\Delta+\omega_1)]}. \tag{66}$$

Similarly,

$$\hat{\mathcal{Q}}_2(\Delta,z,\omega) = \int_{-\infty}^{\infty} d\omega_2\int_{-\infty}^{\infty} d\omega_1 \frac{\hat{\mathcal{S}}(z,\omega-\omega_1+\omega_2)\hat{\mathcal{S}}^*(z,\omega_2)\hat{\mathcal{S}}(z,\omega_1)}{[1/T_2 - i(\Delta+\omega)][1/T_1 - i(\omega_1-\omega_2)][1/T_2 - i(\Delta+\omega_1)]}. \tag{67}$$

For the output field the wave propagation equation Eq. (45) becomes

$$\frac{\partial \mathcal{F}_3(z,\omega)}{\partial z} = \frac{i\omega_0\mathcal{Z}}{2}\left[\mathcal{P}^{(3)}(z,\omega) + \mathcal{P}_3^{(1)}(z,\omega)\right]. \tag{68}$$

where

$$\mathcal{P}_3^{(1)}(z,\omega) = i\, n_D \frac{|\mu_{eg}|^2}{\hbar} \frac{\hat{\mathcal{F}}_3(z,\omega)}{1/T_2 - i(\Delta+\omega)}, \tag{69}$$

is the linear polarisation propagating in the same direction as the output field, $2\boldsymbol{k}_2 - \boldsymbol{k}_1$, and is responsible for output loss due to absorption. Therefore,

$$\frac{\partial \hat{\mathcal{F}}_3(z,\omega)}{\partial z} = \frac{i\omega_0 \mathcal{Z}}{2} \hat{\mathcal{P}}^{(3)}(z,\omega) - \alpha(\Delta,\omega)\hat{\mathcal{F}}_3(z,\omega) = A\hat{\mathcal{Q}}(\Delta,z,\omega) - \alpha(\Delta,\omega)\hat{\mathcal{F}}_3(z,\omega), \tag{70}$$

where $\hat{\mathcal{Q}} = (1/2)(\hat{\mathcal{Q}}_1 + \hat{\mathcal{Q}}_2)$ and

$$A = \frac{\omega_0 \mathcal{Z} n_D |\mu_{eg}|^4}{2\hbar^3} \mathcal{F}_1^*(0,0)\mathcal{F}_2^2(0,0). \tag{71}$$

The solution for the output field at the end of the sample, $z = L$, is

$$\hat{\mathcal{F}}_3(L,\omega) = Ae^{-\alpha(\Delta,\omega)L} \int_0^L dz\, \frac{\hat{\mathcal{Q}}_1(\Delta,z,\omega) + \hat{\mathcal{Q}}_2(\Delta,z,\omega)}{2} e^{\alpha(\Delta,\omega)z}. \tag{72}$$

Using

$$\hat{\mathcal{S}}(z,\omega+\omega_1-\omega_2)\hat{\mathcal{S}}(z,\omega_2)\hat{\mathcal{S}}^*(z,\omega_1) = \hat{\mathcal{S}}(0,\omega+\omega_1-\omega_2)\hat{\mathcal{S}}(0,\omega_2)\hat{\mathcal{S}}^*(0,\omega_1)e^{-[\alpha(\Delta,\omega+\omega_1-\omega_2)+\alpha(\Delta,\omega_2)+\alpha^*(\Delta,\omega_1)]z}, \tag{73}$$

and hence

$$\begin{aligned} &e^{-\alpha(\Delta,\omega)L} \int_0^L dz\, \hat{\mathcal{S}}(z,\omega+\omega_1-\omega_2)\hat{\mathcal{S}}(z,\omega_2)\hat{\mathcal{S}}^*(z,\omega_1)e^{\alpha(\Delta,\omega)z} \\ &= \hat{\mathcal{S}}(0,\omega+\omega_1-\omega_2)\hat{\mathcal{S}}(0,\omega_2)\hat{\mathcal{S}}^*(0,\omega_1)\frac{e^{-\alpha(\Delta,\omega)L} - e^{-[\alpha(\Delta,\omega+\omega_1-\omega_2)+\alpha(\Delta,\omega_2)+\alpha^*(\omega_1)]L}}{\alpha(\Delta,\omega+\omega_1-\omega_2)+\alpha(\Delta,\omega_2)+\alpha^*(\Delta,\omega_1)-\alpha(\Delta,\omega)}, \end{aligned} \tag{74}$$

we have

$$\hat{\mathcal{F}}_3(L,\omega) = AL\,\hat{\mathcal{J}}(\Delta,L,\omega), \tag{75}$$

where $\hat{\mathcal{J}} = (1/2)(\hat{\mathcal{J}}_1 + \hat{\mathcal{J}}_2)$ and

$$\begin{aligned} \hat{\mathcal{J}}_1(\Delta,L,\omega) = \int_{-\infty}^{\infty} d\omega_2 \int_{-\infty}^{\infty} d\omega_1 &\frac{\hat{\mathcal{S}}(0,\omega+\omega_1-\omega_2)\hat{\mathcal{S}}(0,\omega_2)\hat{\mathcal{S}}^*(0,\omega_1)}{[1/T_2 - i(\Delta+\omega)][1/T_1 + i(\omega_1-\omega_2)][1/T_2 + i(\Delta+\omega_1)]} \\ &\times \frac{e^{-\alpha(\Delta,\omega)L} - e^{-[\alpha(\Delta,\omega+\omega_1-\omega_2)+\alpha(\Delta,\omega_2)+\alpha^*(\Delta,\omega_1)]L}}{[\alpha(\Delta,\omega+\omega_1-\omega_2)+\alpha(\Delta,\omega_2)+\alpha^*(\Delta,\omega_1)-\alpha(\Delta,\omega)]\,L}, \end{aligned} \tag{76}$$

$$\begin{aligned} \hat{\mathcal{J}}_2(\Delta,L,\omega) = \int_{-\infty}^{\infty} d\omega_2 \int_{-\infty}^{\infty} d\omega_1 &\frac{\hat{\mathcal{S}}(0,\omega-\omega_1+\omega_2)\hat{\mathcal{S}}^*(0,\omega_2)\hat{\mathcal{S}}^(0,\omega_1)}{[1/T_2 - i(\Delta+\omega)][1/T_1 - i(\omega_1-\omega_2)][1/T_2 - i(\Delta+\omega_1)]} \\ &\times \frac{e^{-\alpha(\Delta,\omega)L} - e^{-[\alpha(\Delta,\omega-\omega_1+\omega_2)+\alpha^*(\Delta,\omega_2)+\alpha(\Delta,\omega_1)]L}}{[\alpha(\Delta,\omega-\omega_1+\omega_2)+\alpha^*(\Delta,\omega_2)+\alpha(\Delta,\omega_1))-\alpha(\Delta,\omega)]\,L}. \end{aligned} \tag{77}$$

D. Effect of inhomogeneous broadening

To take into account the effect of inhomogeneous broadening the output field in Eq. (75) and thus $\hat{\mathcal{J}}$ of Eqs. (76) and (77) has to be averaged over the Gaussian distribution of Eq. (25)

$$\hat{\mathcal{J}}(\Delta,L,\omega) \to \langle\hat{\mathcal{J}}\rangle(\Delta,L,\omega) = \int_{-\infty}^{\infty} d\delta\, g(\delta)\hat{\mathcal{J}}(\Delta-\delta,L,\omega), \tag{78}$$

and the output field of Eq. (75) now becomes

$$\hat{\mathcal{F}}_3(L,\omega) = AL\,\langle\hat{\mathcal{J}}\rangle\,(\Delta,L,\omega) = \frac{\omega_0 \mathcal{Z} n_D |\mu_{eg}|^4 L}{2\hbar^3}\mathcal{F}_1^*(0,0)\mathcal{F}_2^2(0,0)\,\langle\hat{\mathcal{J}}\rangle\,(\Delta,L,\omega). \tag{79}$$

Before calculating the input and output energy, we first note that the pump and the rephasing beam in our experiment has a Gaussian radial profile, i.e., the field $\mathcal{F}_{1,2}$ decreases with the distance from the center of the beam, ρ, through the factor

$$\mathcal{W}(\rho) = e^{-\rho^2/2\rho_0^2}, \tag{80}$$

where ρ_0 is the r.m.s. radius of the electric field profile of the beam, and is related to the r.m.s radius of the intensity profile in the Main Text, r_0, by $\rho_0 = \sqrt{2}r_0$. Since the four wave mixing output field is proportional to $\mathcal{F}_1^*(0,0)\mathcal{F}_2^2(0,0)$, its radial profile is $\mathcal{W}^3(\rho)$.

To calculate the energy one has to integrate the intensity over time and then calculate the spatial integration over the radial profile. The input energy at $z=0$ is

$$\begin{aligned} E_{1,2} &= \frac{1}{2\mathcal{Z}}\int_0^\infty 2\pi\rho\,d\rho\int_{-\infty}^\infty dt\,|\mathcal{F}_{1,2}(0,t)\mathcal{W}(\rho)|^2 = \frac{1}{2\mathcal{Z}}|\mathcal{F}_{1,2}(0,0)|^2\int_0^\infty 2\pi\rho\,d\rho\,|\mathcal{W}(\rho)|^2\int_{-\infty}^\infty dt\,|\mathcal{S}(0,t)|^2 \\ &= \frac{1}{2\mathcal{Z}}|\mathcal{F}_{1,2}(0,0)|^2\pi\rho_0^2\sqrt{\pi}\tau, \end{aligned} \tag{81}$$

and the output energy at $z=L$ is

$$\begin{aligned} E_3 &= \frac{1}{2\mathcal{Z}}\int_0^\infty 2\pi\rho\,d\rho|\mathcal{W}^3(\rho)|^2\int_{-\infty}^\infty dt|\mathcal{F}_3(L,t)|^2 \\ &= \frac{1}{2\mathcal{Z}}\frac{\pi\rho_0^2}{3}\int_{-\infty}^\infty 2\pi\,d\omega\,|\mathcal{F}_3(L,\omega)|^2. \end{aligned} \tag{82}$$

Using Eq. (79) we obtain

$$E_3 = \frac{\mathcal{Z}}{2}\left(\frac{\omega_L n_D|\mu_{eg}|^4 L}{2\hbar^3}\right)^2|\mathcal{F}_1^*(0,0)\mathcal{F}_2^2(0,0)|^2\frac{\pi\rho_0^2}{3}\int_{-\infty}^\infty 2\pi\,d\omega\,|\langle\hat{\mathcal{J}}\rangle(\Delta,L,\omega)|^2. \tag{83}$$

The critical energy discussed in the Main Text is

$$E_c = \sqrt{\frac{E_1E_2^2}{E_3}} = \left(\frac{\hbar^3}{\mathcal{Z}^2\omega_L n_D|\mu_{eg}|^4 L}\right)\sqrt{3}\pi\rho_0^2\,(\sqrt{\pi}\tau)^{3/2}\left(\int_{-\infty}^\infty 2\pi\,d\omega\,|\langle\hat{\mathcal{J}}\rangle(\Delta,L,\omega)|^2\right)^{-1/2}. \tag{84}$$

From Eq. (26) we have

$$\frac{n_D|\mu_{eg}|^4}{\hbar^3} = \frac{\epsilon_0|\chi^{(3)}|}{T_1T_2^2|\eta|} = \frac{|\chi^{(3)}|}{T_1T_2^2|\eta|\mathcal{Z}_0 c}. \tag{85}$$

Substituting this into the formula for E_c, we obtain

$$\begin{aligned} E_c &= \left(\frac{\mathcal{Z}_0 c\,T_1T_2^2|\eta|}{\mathcal{Z}^2\omega_L|\chi^{(3)}|L}\right)\sqrt{3}\pi\rho_0^2\,(\sqrt{\pi}\tau)^{3/2}\left(\int_{-\infty}^\infty 2\pi\,d\omega\,|\langle\hat{\mathcal{J}}\rangle(\Delta,L,\omega)|^2\right)^{-1/2} \\ &= \kappa I_c\rho_0^2\tau, \end{aligned} \tag{86}$$

where the critical intensity is defined as

$$I_c = \frac{\mathcal{Z}_0 c}{\mathcal{Z}^2\omega_0|\chi^{(3)}|L} = \frac{n^2\lambda_0}{2\pi L\mathcal{Z}_0|\chi^{(3)}|} \tag{87}$$

with $\lambda_0 = 2\pi c/\omega_0$, the carrier wavelength of the laser, and

$$\kappa = \sqrt{3}\pi^{7/4}|\eta|T_1T_2^2\left(\frac{\tau}{\int_{-\infty}^\infty 2\pi\,d\omega\,|\langle\hat{\mathcal{J}}\rangle(\Delta,L,\omega)|^2}\right)^{1/2}. \tag{88}$$

Note that the integral in the denominator has the dimension of [time]^7 and therefore κ is dimensionless.

It is useful to express the critical energy in a form that is convenient for a comparison with its value in the ideal case when the input pulses are infinitely long, $\tau \gg T_1, T_2$, and when the loss due to absorption can be neglected. In this limit $\alpha L \to 0$, and moreover $\hat{S}(0,\omega)$ is a very sharp distribution around $\omega = 0$. Setting all the frequencies in the denominators of Eqs. (76) and (77) to zero we have

$$\begin{aligned}\hat{\mathcal{J}}_1^{(\infty)}(\Delta, L, \omega) &= \frac{1}{(1/T_2 - i\Delta)(1/T_1)(1/T_2 + i\Delta)} \int_{-\infty}^{\infty} d\omega_1 \int_{-\infty}^{\infty} d\omega_2\, \hat{\mathcal{S}}(0, \omega - \omega_1 + \omega_2)\hat{\mathcal{S}}(0,\omega_2)\hat{\mathcal{S}}^*(0,\omega_1) \\ &= \frac{1}{(1/T_2 - i\Delta)(1/T_1)(1/T_2 + i\Delta)} \frac{\tau e^{-\frac{1}{6}\tau^2\omega^2}}{\sqrt{6\pi}}. \end{aligned} \tag{89}$$

Similarly

$$\hat{\mathcal{J}}_2^{(\infty)}(\Delta, L, \omega) = \frac{1}{(1/T_2 - i\Delta)(1/T_1)(1/T_2 - i\Delta)} \frac{\tau e^{-\frac{1}{6}\tau^2\omega^2}}{\sqrt{6\pi}}. \tag{90}$$

Then

$$\hat{\mathcal{J}}^{(\infty)}(\Delta, L, \omega) = \frac{\hat{\mathcal{J}}_1^{(\infty)}(\Delta, L, \omega) + \hat{\mathcal{J}}_2^{(\infty)}(\Delta, L, \omega)}{2} = \frac{i\,T_1 T_2^2}{(1+\Delta^2 T_2^2)(i + \Delta T_2)} \frac{\tau e^{-\frac{1}{6}\tau^2\omega^2}}{\sqrt{6\pi}}, \tag{91}$$

and after averaging over the distribution of the inhomogeneous broadening

$$\langle \hat{\mathcal{J}} \rangle^{(\infty)}(\Delta, L, \omega) = T_1 T_2^2 i\,\eta \frac{\tau e^{-\frac{1}{6}\tau^2\omega^2}}{\sqrt{6\pi}}, \tag{92}$$

so

$$\int_{-\infty}^{\infty} 2\pi\, d\omega |\langle \hat{\mathcal{J}} \rangle^{(\infty)}(\Delta, L, \omega)|^2 = \sqrt{\frac{\pi}{3}} T_1^2 T_2^4 |\eta|^2 \tau. \tag{93}$$

Substituting this into Eq. (88) and then Eq. (86) we obtain

$$E_c^{(\infty)} = 3^{3/4}\pi^{3/2} I_c \rho_0^2 \tau. \tag{94}$$

Thus we can express the critical energy in Eq. (86) as

$$E_c = E_c^{(\infty)} f = 3^{3/4}\pi^{3/2} I_c \rho_0^2 \tau f, \tag{95}$$

where

$$f = \left(\frac{\pi}{3}\right)^{1/4} |\eta| T_1 T_2^2 \left(\frac{\tau}{\int_{-\infty}^{\infty} 2\pi\, d\omega |\langle \hat{\mathcal{J}} \rangle(\Delta, L, \omega)|^2} \right)^{1/2}. \tag{96}$$

The factor f describes the effect of finite duration of the laser pulses, as well as the loss of both the input and output pulses due to absorption. It reduces to unity in the ideal limit. For the off resonant laser frequencies in our experiment we found that the loss is negligible, i.e., $\alpha(\Delta, \omega)L \ll 1$, but f differs from unity due to the short duration of the pulses. For the resonant laser frequencies the loss due to absorption is strong, thus $\langle \hat{\mathcal{J}} \rangle(\Delta, L, \omega)$ is very small, and f is very large.

Finally, we replace $\tau = \sqrt{2} t_0$ and $\rho_0 = \sqrt{2} r_0$ and arrive at

$$E_c = 3^{3/4}(2\pi)^{3/2} I_c \rho_0^2 \tau f = \frac{3^{3/4}\sqrt{2\pi} n^2 \lambda_0 r_0^2 t_0 f}{\mathcal{Z}_0 |\chi^{(3)}| L}, \tag{97}$$

which is the formula we use to extract $\chi^{(3)}$ from the experimental measurement of E_c, as mentioned in the Methods section of the Main Text.

We see from Eqs. (76), (77) and (78) that $\langle \hat{\mathcal{J}} \rangle(\Delta, L, \omega)$ is a three dimensional integral, and therefore the denominator in the large bracket of f is a four dimensional integral. This can be computed efficiently with Monte-Carlo methods. Our calculation in Mathematica is available at https://github.com/lehnqt/4WM/. The results for f in the conditions shown in Table I of the Main Text are given in the same table. Note that for this calculation we took $\Gamma \approx 1/\sqrt{2\ln(2)} T_2^*$ since the lines are inhomogeneously broadened. We check that in the lossless and long pulse limit our numerical calculation gives $f \approx 1$.

E. Analytical approximations for short pulses

1. Far off resonance

Far away from resonance the loss is negligible because $\alpha(\Delta,\omega)L \ll 1$. In addition, the detuning is much larger than the width of the inhomogeneous broadening, $\Delta \gg \Gamma$, as well as the spectral width of the laser, $\Delta \gg 1/\tau$, so the effect of the inhomogeneous broadening can also be ignored. Moreover, the detuning of the central laser frequency is much larger than the width of the line, $\Delta \gg 1/T_2, 1/T_1$, and for short pulses the duration satisfies $\tau \ll T_1, T_2$. This leads to simplification in the expression of the output field.

We use the following approximation:

$$\int_0^\infty e^{-i\Delta t - t/T_2}\mathcal{S}(t) = \frac{1}{i\Delta + 1/T_2}[\mathcal{S}(0) - \mathcal{S}(\infty)], \tag{98}$$

To prove this consider the integration from $t = 0$ to $t = 2\pi/\Delta$ which is the very short period of the oscillating factor. In this interval $\mathcal{S}(t)$ changes very little and we can use its Taylor expansion to first order

$$\mathcal{S}(t) \approx \mathcal{S}(0) + \mathcal{S}'(0)t. \tag{99}$$

Then

$$\int_0^{2\pi/\Delta} e^{-i\Delta t - t/T_2}\mathcal{S}(t) \approx \int_0^{2\pi/\Delta} e^{-i\Delta t - t/T_2}\left[\mathcal{S}(0) + \mathcal{S}'(0)t\right] \tag{100}$$

has a simple analytical result. Using $\Delta T_2 \gg 1$ and the fact that the duration of $\mathcal{S}$ is much smaller than T_2 one can show that this analytical result can be approximated by

$$\int_0^{2\pi/\Delta} e^{-i\Delta t - t/T_2}\left[\mathcal{S}(0) + \mathcal{S}'(0)t\right] \approx \frac{-1}{i\Delta + 1/T_2}\mathcal{S}'(0)\frac{2\pi}{\Delta} \approx \frac{1}{i\Delta + 1/T_2}[\mathcal{S}(0) - \mathcal{S}(2\pi/\Delta)], \tag{101}$$

and Eq. (98) follows from applying this repeatedly until infinity.

Now we evaluate $\mathcal{Q}_{1,2}(\Delta, z, t)$ in Eq. (61) (we drop the z variable because when the loss is negligible $\mathcal{S}$ and hence $\mathcal{Q}_{1,2}$ are independent of z)

$$\int_{-\infty}^{t_2} dt_1 e^{-(t_2-t_1)/T_2} e^{i\Delta t_1}\mathcal{S}^*(t_1) = \int_0^\infty dt_1' e^{-i\Delta t_1' - t_1'/T_2}\mathcal{S}^*(t_2 - t_1')e^{i\Delta t_2} = \frac{1}{1/T_2 + i\Delta}S^*(t_2)e^{i\Delta t_2},$$

and then

$$\frac{1}{1/T_2 + i\Delta}\int_{-\infty}^{t_3} dt_2 e^{-(t_3-t_2)/T_1}\mathcal{S}(t_2)e^{-i\Delta t_2}S^*(t_2)e^{i\Delta t_2} = \frac{1}{1/T_2 + i\Delta}\int_0^\infty dt_2' e^{-t_2'/T_1}|S(t_3 - t_2')|^2 = \frac{1}{1/T_2 + i\Delta}\mathcal{Y}(t_3),$$

where

$$\mathcal{Y}(t_3) = \int_0^\infty dt_2' e^{-t_2'/T_1}|S(t_3 - t_2')|^2. \tag{102}$$

Note that $S(t_3 - t_2')$ is non-negligible only when t_2' is within 3τ around t_3 and since $\tau \ll T_1$ the exponential factor e^{-t_2'/T_1} changes very little over this interval, hence

$$\mathcal{Y}(t_3) \approx e^{-t_3/T_1}\int_0^\infty dt_2'|S(t_3 - t_2')|^2 = e^{-t_3/T_1}\frac{\sqrt{\pi}\tau}{2}\left[1 + \mathrm{Erf}\left(\frac{t_3}{\tau}\right)\right]. \tag{103}$$

And finally,

$$\begin{aligned}\frac{e^{i\Delta t}}{1/T_2 + i\Delta}\int_{-\infty}^{t} dt_3 e^{-(t-t_3)/T_2}e^{-i\Delta t_3}\mathcal{S}(t_3)\mathcal{Y}(t_3) &= \frac{e^{i\Delta t}}{1/T_2 + i\Delta}\int_0^\infty dt_3' e^{i\Delta t_3' - t_3'/T_2}S(t - t_3')Y(t - t_3')e^{-i\Delta t} \\ &= \frac{1}{1/T_2 + i\Delta}\frac{1}{1/T_2 - i\Delta}S(t)Y(t),\end{aligned}$$

where we have used Eq. (98) again. Thus,

$$\mathcal{Q}_1(\Delta,t)=\frac{T_2^2}{(1-i\Delta T_2)(1+i\Delta T_2)}\mathcal{S}(t)\mathcal{Y}(t). \tag{104}$$

Similarly

$$\mathcal{Q}_2(\Delta,t)=\frac{T_2^2}{(1-i\Delta T_2)^2}\mathcal{S}(t)\mathcal{Y}(t), \tag{105}$$

so

$$Q(\Delta,t)=\frac{T_2^2}{(1-i\Delta T_2)\,(1+\Delta^2T_2^2)}S(t)Y(t)=i\,\eta T_2^2S(t)Y(t), \tag{106}$$

where the last step follows from Eq. (28). Again, since $\mathcal{S}(t)\mathcal{Y}(t)$ is non-negligible only in the interval $[-3\tau,3\tau]$ and $\tau\ll T_1$ we can neglect the exponential factor in $\mathcal{Y}(t)$ and hence

$$Q(\Delta,t)\approx i\,\eta T_2^2S(t)Y(t)=i\,\eta T_2^2e^{-t^2/2\tau^2}\frac{\sqrt{\pi}\tau}{2}\left[1+\mathrm{Erf}\left(\frac{t}{\tau}\right)\right]. \tag{107}$$

where Erf is the error function. The last step follows from the Gaussian form of $\mathcal{S}$ in Eq. (48).

Without loss the function $\hat{\mathcal{J}}(\Delta,\omega)$ in the denominator of the factor f is just $\hat{\mathcal{Q}}(\Delta,\omega)$, the Fourier transform of $\mathcal{Q}(\Delta,t)$, thus

$$\int_{-\infty}^{\infty}2\pi\,d\omega|\hat{\mathcal{J}}(\Delta,\omega)|^2=\int_{-\infty}^{\infty}dt\,|\mathcal{Q}(\Delta,t)|^2=|\eta|^2T_2^4\tau^2\frac{\pi}{4}\int_{-\infty}^{\infty}dt\,e^{-t^2/\tau^2}\left[1+\mathrm{Erf}\left(\frac{t}{\tau}\right)\right]^2=\frac{\pi^{3/2}}{3}T_2^4\tau^3|\eta|^2, \tag{108}$$

and thus the factor f in Eq. (96) becomes

$$f=\frac{3^{1/4}}{\sqrt{\pi}}\frac{T_1}{\tau}=\frac{3^{1/4}}{\sqrt{2\pi}}\frac{T_1}{t_0}. \tag{109}$$

Thus, the critical energy of Eq. (97) is related to $\chi^{(3)}$ by the simple relation

$$E_c=\frac{3n^2\lambda_0r_0^2t_0^2}{\mathcal{Z}_0T_1|\chi^{(3)}|L}, \tag{110}$$

2. *On resonance*

For completeness we provide the analytical solution for resonant cases where the detuning of the central frequency is small compared with the spectral width of the laser, i.e., $\Delta,1/T_{\mathrm{inh}}\ll1/\tau$, and for thin samples where the loss due to absorption can be neglected. When the pulse duration is short so that $\tau\ll T_1,T_2$ we can approximate $\mathcal{S}$ as a delta function with area $s=\sqrt{2\pi}\tau$, which is the area under $\mathcal{S}$, to evaluate the integrals of $Q_{1,2}(\Delta,t)$.

$$\int_{-\infty}^{t_2}dt_1e^{-(t_2-t_1)/T_2}\tilde{\mathcal{S}}^*(\Delta,t_1)=\int_{-\infty}^{t_2}dt_1e^{-(t_2-t_1)/T_2}e^{i\Delta t_1}s\delta(t_1)=s\,e^{-t_2/T_2}\Theta(t_2),$$

where $\Theta(t_2)$ is the step function. Then

$$\int_{-\infty}^{t_3}dt_2e^{-(t_3-t_2)/T_1}\tilde{\mathcal{S}}(\Delta,t_2)s\,e^{-t_2/T_2}\Theta(t_2)=\int_0^{t_3}dt_2e^{-(t_3-t_2)/T_1}e^{-t_2/T_2}e^{-i\Delta t_2}s^2\delta(t_2)=\frac{s^2}{2}e^{-t_3/T_1}\Theta(t_3),$$

and

$$\mathcal{Q}_1(\Delta,t)=\int_{-\infty}^{t}dt_3e^{-(t-t_3)/T_2}\tilde{\mathcal{S}}(\Delta,t_3)\frac{s^2}{2}e^{-t_3/T_1}\Theta(t_3)=\int_0^{t}dt_3e^{-(t-t_3)/T_2}e^{-t_3/T_1}e^{-i\Delta t_3}\frac{s^3}{2}\delta(t_3)=\frac{s^3}{4}e^{-t/T_2}\Theta(t), \tag{111}$$

and similarly we find that $\mathcal{Q}_2(\Delta, t) = \mathcal{Q}_1(\Delta, t)$, so

$$\int_{-\infty}^{\infty} dt\, \mathcal{Q}^2(\Delta, t) \approx \int_0^{\infty} dt\, \frac{s^6}{16} e^{-2t/T_2} = \frac{\pi^3}{4}\tau^6 T_2. \tag{112}$$

Therefore,

$$f = \frac{2|\eta| T_1 T_2^{3/2}}{(3\pi^5)^{1/4}\tau^{5/2}} = \frac{|\eta| T_1 T_2^{3/2}}{(6\pi^5)^{1/4} t_0^{5/2}}, \tag{113}$$

and the critical energy is related to $\chi^{(3)}$ by

$$E_c = \sqrt{3}\left(\frac{2}{\pi^3}\right)^{1/4} \frac{|\eta| n^2 \lambda_0 r_0^2 T_1}{\mathcal{Z}_0 |\chi^{(3)}| L} \left(\frac{T_2}{t_0}\right)^{3/2}. \tag{114}$$

As before, if the line is homogeneous then $\eta \approx -i$ (Eq. (30)) and if the inhomogeneous broadening is large $\eta \approx -i\sqrt{\pi \ln 2}\, T_{\text{inh}}/2T_2$ (Eq. (29)).

Finally, we stress that the approximate formulae, Eq. (109) for the off resonant and Eq. (113) for the on resonant case, are useful for predicting how f varies with T_1, T_2 and t_0 in the short pulse limit. They can also be used for an order of magnitude estimation. However, comparison with exact numerical results shows that a precise agreement requires including higher order terms in Eq. (98) for the off resonant case, and moving beyond the Dirac delta approximation to take into account the shape of the Gaussian for the on resonant case.

[1] J. F. Reinertsen, *Simulation of Ultrafast Pump-Probe Measurements for Semiconductors*, Master's thesis, Norwegian University of Science and Technology, Trondheim (2012).

[2] R. W. Boyd, *Nonlinear Optics* (Academic Press, 2008) chapter 2.
